# The Importance of Parents’ Income and Education Level in Relation to Their Preschool Children’s Activity Level at Leisure

**DOI:** 10.3390/children8090733

**Published:** 2021-08-26

**Authors:** Oda Malmo, Karin Kippe, Pål Lagestad

**Affiliations:** Department of Teacher Education, Nord University, 7600 Levanger, Norway; Oda.Andvik.Malmo@stjordal.kommune.no (O.M.); pal.a.lagestad@nord.no (P.L.)

**Keywords:** preschool children, physical activity, health, parents, socio-economic status

## Abstract

Previous research indicate that socioeconomic status positively corresponds with adults’ and adolescents’ physical activity levels. This study investigated the relationship between parents’ education and income, and preschool children’s physical activity level. A total of 244 Norwegian preschool children aged four to six and their parents were enrolled in the study. The children wore an Actigraph GT1M accelerometer for seven consecutive days to measure their physical activity level. Parents completed a questionnaire that provided information about their education level and income level. To examine the relationship between the parents’ education and income and their children’s physical activity level at leisure, the Kruskal-Wallis H test was conducted. The results revealed that neither mothers’ nor fathers’ education level or income, were associated with their children’s minutes in moderate to vigorous physical activity (MVPA) at leisure. The preschool curriculum of Norway may be one explanation why socioeconomic status was not linked to physical activity in this study. Another possibility is that this study was limited to full-time students with two parents. More research is needed to determine whether parent income or education is linked to physical activity among more diverse or older children in Norway.

## 1. Introduction

Despite the health benefits of physical activity [1,2,3,4,5,6,7,8,9], the levels of physical activity have been found to decrease with age in adolescence [10,11,12]. Two Norwegian studies [13,14] found that approximately 80% of four to six year old children met the global recommendations of 60 min in vigorous physical activity (MVPA) daily, while 56.8% of the 15-year-old girls and 41.9% of the boys were insufficiently physically active [13]. A cross-sectional study report that the total amount of physical activity decreases by an average of 4.2% each year from the age of 5 to 18 [15]. Such findings have become a cause of great concern, taking into consideration that not being sufficient physical active, have been identified as a leading risk factor for global mortality [16], and that inactivity among children and youth has been linked with lower physical activity levels in later life [17]. Consequently, understanding the social determinants and factors that are associated with young people’s physical activity habits has become a topic of great interest, especially children in preschool age. This study investigates the relationship between parents’ income and education level, and their children’s physical activity.

Some studies have shown that mothers physical activity level is linked to their children’s physical activity [18,19,20], and there has been a need for investigating what adult characteristics that are associated with physical activity. Studies have revealed that adults with higher socioeconomic status tend to be more physically active compared to those with lower socioeconomic status [21]. Studies investigating the association of parental education level and children’s total physical activity refers to various findings [18,22,23]. This is in line with studies investigating the association between parental income and children’s total physical activity [24,25]. Lu et al. [19] and O’Donoghue [26] did not find an association between preschool children’s physical activity level and parental socioeconomic status. In contrast, studies investigating the association between parental education and income, and children’s total physical activity level seem to positively correspond [27,28], indicating that children’s physical activity level increase with rising household education and income. The relationship between children’s physical activity and their parents’ education and income is theoretically explained in relation to parents’ habits, and how they play a significant part in young children’s physical activity habits [18,29,30]. Research has shown that physical activity levels among adults, are positively correlated with education and income [21]. Young children are mostly in relationship with significant others such as family members, whereas children internalize and take possession of their family’s attitudes, values and actions through interaction, closeness and identification [31,32,33,34], and thereby parents transfer inherited physical capital to their children. Preschools and public health authorities have a key role in connection with information and guidance of the parents regarding the importance of physical activity. In collaboration, preschool, health authorities and parents can prevent health inequalities through information about the health benefits of being physically active and facilitating physical activity in the children’s neighborhood at leisure. This can be safe areas without busy roads, and green areas where families can go for walks and trips. Areas for physical activity in the local environment lowers the threshold for physical activity, and can contribute to more people becoming physically active.

Additionally, previous research indicates that the relationship between socioeconomic status and children’s physical activity level seems to be related to how physical activity is measured. Existing studies reporting a positive relationship between socioeconomic status and children’s physical activity, tend to use questionnaire as the only method to gather information on physical activity [23,35,36,37,38,39]. An interesting aspect is that studies reporting no association [18] or a negative association [22,24] between parents’ socioeconomic status and children’s physical activity use either accelerometers to measure physical activity or a combination of questionnaire and physical activity recall. Considering the fact that numerous researchers seem to agree that calorimetric validated accelerometers are the most promising method for measuring physical activity in free-living situations [40,41], it is reasonable to argue that studies conducted using this method are more reliable.

Due to results in studies conducted on children in preschool age are conflicted, reporting both no association [18], a positive association [42] and a negative association [22] between socioeconomic status and preschool children’s physical activity, this study is highly relevant to gain more knowledge about the determinants of socioeconomic status and physical activity in early childhood. It is important to encourage all children to be physically active regardless of their parents’ socioeconomic status. This may help all children to develop good physical activity habits later in life. Therefore, it is of great importance to focus on the primary socialization where children’s physical habits develop. Ennis [32] has pointed out that motor skills is crucial for realizing the goal of lifelong interest in physical activity. Motor skills probably contribute to children participating in varied physical activities, showing greater endurance, and engaging in physically demanding activities [32]. Positive physical activity habits are important to establish before the children’s natural urge for physical activity disappears. By stimulating all children to develop positive attitudes towards physical activity, for example in preschool, we can prevent health inequalities. The Norwegian preschool framework plan [43] establishes that preschools shall, in cooperation and understanding with the children’s parents, take responsibility for the children’s need for care and play, and promote learning and education as a basis for versatile development. According to the framework plan, the preschools must be a daily arena for physical activity, and promote the children’s joy in physical activity and motor development. The aim of this study is to explore the relationship between parental education and income, and preschool children’s physical activity level by examining the research question: “How are parental education and income related to their children’s physical activity level at leisure?”

## 2. Material and Methods

The present study utilizes already existing data that were collected for a larger Ph.D. research project, where data regarding the relationship between income, education and children’s activity level at leisure were unpublished [14,44]. Only accelerometer data and questionnaire data from this project were of relevance to examine the research question in the present study. Accelerometer data provides an objective measure of intensity, frequency and duration of physical activity [13,41,45], whilst questionnaires is used to measure education level and income. The study was approved by NSD (Norwegian Centre for Research Data, ref. 52221) (28 February 2021), which is an ethics committee.

### 2.1. Participants and Procedure

A total of 13 preschools in the northern part of Troendelag, a county located in central Norway, were randomly selected to participate in the study. The county consists of both cities and more rural areas. All of the 13 preschools in the study were located in the outskirts of the city. The eligibility criteria of this study were that all children had to be full-time pre-schoolers, and 4–6 years of age. Out of the 13 preschools, 364 children met the first criteria, of which 289 children volunteered to participate in the study by the approval of their primary guardian. Out of these, 244 children (125 boys and 119 girls) had valid accelerometer data and parents who had completed the parental questionnaire—constituting a response rate of 67%. In total, 198 mothers and 153 fathers participated in the study. There were two fathers who did not declare income. To be included in the study, the children had to be from two-parent families. This could be unfortunate, as some families only had one parent fill out the questionnaire and some children are raised without two parents.

Prior to the data collection and signing of the consent forms, parents and preschool staff were given written and oral information about the procedures and ethical standards for testing related to sports science. Among other things, this included information about the participants right to decline to participate, and their right to withdraw from the research once it has started. Accelerometer data and questionnaire data were collected during a consecutive five-week period in 2017—from the middle of May until the end of June.

### 2.2. Accelerometry

Physical activity was measured with standardized procedures used in a large population study among children in Norway [13]. Physical activity data were objectively collected using Actigraph GT1M accelerometers (ActiGraph, Fort Walton Beach, FL, USA). Accelerometers have emerged to be a valid and reliable tool for measuring daily physical activity and intensity among children [40,45,46,47,48,49]. The ActiGraph GT1M accelerometer measures acceleration in the horizontal and vertical direction, but the vertical direction is used. The acceleration creates electrical voltage in proportion to the acceleration. This voltage is digitized 30 times per second (30 Herz) and filters with a frequency sensitivity of 0.25–2.5 Herz, that corresponds to human movement. Raw accelerometer data output is referred to as counts per minute (CPM). Counts describe the accelerations the accelerometer has been exposed to during defined epochs (time intervals), divided by the number of minutes registered as wear time [13,50]. In order to describe users’ physical activity, the number of counts were classified into four categories (i.e., sedentary, light, moderate and vigorous physical activity) based on already validated count cutoffs from a Norwegian population study [13]. Activity that produced 100–1999 CPM was interpreted as light physical activity, whereas activity with less than 100 CPM was defined as sedentary. Moderate activity required 2000–5998 CPM, while activities that produced more than 5998 CPM was interpreted as vigorous physical activity [13].

Subjects were required to wear the accelerometers for seven consecutive days, which is recommended by numerous researchers [51,52,53,54]. To ensure that all subjects wore the device throughout the period, a text message was sent to the parents each morning, reminding them to have their child wear the accelerometer. The parents were also informed about its use and that the accelerometer had to be placed on the right hip. The subjects had to wear the accelerometer for all waking hours, except during showering or other water activities. The accelerometers were set to start recording at 06.00 a.m. the day after they were distributed and put on, as an attempt to counteract the Hawthorne effect [55].

At the end of the data collection, the accelerometers were retrieved from the subjects and the data were downloaded to the ActiLife v6 13.3 Software program (ActiGraph LLC, Pensacola, FL, USA). The accelerometers recorded data in 10 s epochs, providing the number of counts for every 10 s for the 7-day period. Subjects were required to have at least two valid days in order to be included in the analyses. For data to be considered valid, at least 480 min of daily recorded activity was required. Additionally, sequences of 20 min or more with zero counts were interpreted as non-wearing time, and were therefore, removed. Due to instructions saying that subjects were not to wear accelerometers during sleep, data between 00:00 and 05.59 a.m. were also excluded. Wear-time was categorized as the following variables: preschool hours (08:00 a.m.–03:29 pm), leisure time on weekdays (06:00 a.m.–07:59 a.m. and 03.30 p.m.–11:59 p.m.) and weekends (06:00 a.m.–11:59 p.m.). These operationalizations were made based on feedback from parents and preschool staff, who identified these times as time spent in preschool and leisure, respectively.

### 2.3. Questionnaires

The parents of the participating children were given a questionnaire that they were asked to complete to provide relevant information, including their education level and income level. We also asked the parents to report their physical activity habits. The questionnaire was designed on the basis of already validated and reliability-tested questions from studies of Hansen et al. [56] and HUNT3 [57]. Parental education level was measured in accordance with the Norwegian education system, where the reply options were “less than seven years at primary school”, “7–10 years at primary school”, “high school, vocational subjects”, “high school, specialization in general studies”, “1–3 years at college and/or university” and “college and/or university education for more than four years”. Parental income level was based on their reported gross income per year (including earnings such as capital gains, pensions, child benefits and other social benefits). The response options were “10,000–20,000”, “30,000–40,000”, “50,000–60,000”, 70,000–80,000”, 90,000–1,000,000” and >100,000 Euros. According to Statistics Norway [58], the average full-time monthly salary in Norway is approximately 50,000 NOK (4800 Euros). Men earn, on average, a little more than woman, 51,600 NOK (5200 Euros) for men against 45,200 NOK (4560 Euros) pr. month for women. Prior to the data collection, the questionnaire was pre-tested by 10 parents of 4 to 6-year-old preschoolers in a preschool that did not participate in the study. Parent physical activity was examined using the following questions: “How many hours in the last week have you been in physical activity (defined as physical activity where you got warm, sweat and increased respiratory rate) at home or in connection with home? (Report only physical activities that last for at least 10 min). The response options were “None, <1 h, 1–2 h, >4 h”. The parents were also asked: “When your child is in physical activity in leisure time, how much of this time (0–100%)”.

### 2.4. Data Analysis

The accelerometer data and the data collected in the parental questionnaire were analyzed in SPSS Statistics version 26 (IBM SPSS, Chicago, IL, USA). Descriptive characteristics will be presented with mean and standard deviation. Correlations between the children’s physical activity and their mothers and fathers weekly physical activity was performed using Spearman’s correlation analyses. Independent t-tests were used to examine differences in MVPA between boys and girls. Furthermore, education level and income were reorganized in three categories. Education = 1 (up to 10 years at primary school), 2 (high school), 3 (college and/or university). Income = 1 (up to 499,000 Norwegian kroner), 2 (500,000–699,000 Norwegian kroner), 3 (>699,000 Norwegian kroner and more). The Kruskal-Wallis H test was conducted in order to examine the association between the independent variables (mothers/fathers’ education/income) and the dependent variable (children’s MVPA level at leisure). Mann–Whitney U tests were used as follow up analyses between the three groups, using Bonferroni corrections.

## 3. Results

An initial correlation analysis showed no correlation (*p* > 0.05) between the children’s physical activity, and their mothers and fathers weekly physical activity in moderate intensity. The mothers estimated an average of 50.8% (SD 22) of the time where they participated with the child in the children’s physical activity at leisure, while the father’s estimations was 44% (SD 23.8). The results show that there were no significant differences between boys and girls MVPA on weekdays and weekends. Mean physical activity in MVPA was 29 min. for the three-year old children, 32 min. for the four-year old children, 36.5 min. for the five-year old children and 37 min. for the six-year old children on weekdays. The descriptive characteristics of children’s MVPA level at leisure are shown in Table 1.

The descriptive characteristics of children’s MVPA level at leisure, according to the independent variables: mothers’ education level and income is shown in Table 2. The results show that mothers education level is not significantly related to the children’s physical activity level in MVPA at leisure (*p* > 0.05). Statistical analyses show that the mothers’ income is related children’s physical activity level in MVPA at leisure significantly, but at a borderline level (Z = 6.3, *p* = 0.043). However, follow up analyses with Bonferroni corrections show no significant differences between the three groups of income.

The results show that neither the fathers’ education level nor income are related to the children’s physical activity level in MVPA at leisure (*p* > 0.05). The descriptive characteristics of children’s MVPA level at leisure, according to the independent variables: fathers’ education level and income is shown in Table 3.

## 4. Discussion

The results point towards no significant relationship between preschool children’s activity level at leisure, and their parents’ income and education level. At the same time, this is an interesting finding as it might imply that most children of preschool age are initially physically active, as suggested in another study [58], and that socialization mechanisms may be more prominent later on. Previous studies that have examined the relationship between parental education and/or income, and preschool children’s (<6 years) physical activity level, have conflicting findings [18,22,42], while findings from studies conducted on adolescents above preschool age are more consistent [23,25,35,36,37,38,59], which indicate that the effect of parental socioeconomic status might become more prominent in school age.

For adults, research has demonstrated that those with higher socio-economic status tend to be more physically active compared to those with lower socioeconomic status [21]. Furthermore, several researchers claim that adults have a positive impact on preschool children’s physical activity level [30,60], especially those who shows enjoyment being in physical activity [21]. This indicates that adults’ behavior and actions are important factors associated with young children’s activity level. Research implies that parents’ attitudes, values and actions are especially important for their children’s socialization process in young age, as children take possession of their family’s attitudes, values and actions through interaction, closeness and identification [31]. Regarding this, it is suggested by Goldfield et al. [30] and WHO [29] that physical activity should be prompted within the first five years of living because children’s activity patterns are more easily influenced and open to changes and adoptions. The children’s age in our study (four to six) is a period where they are mostly in relationship with significant others, such as family members, whereas children internalize and take possession of their family’s attitudes, values and actions through interaction, closeness and identification [31]. In some studies, the children’s mothers’ physical activity level has been linked to their children’s physical activity [18,19,20]. It is of great importance that all parents are encouraged to be physically active with their children to strengthen the children’s health. Parents must also be guided on how to facilitate their children’s physical activity. Furthermore, it is also essential that parents receive information about the health benefits of a physically active lifestyle. Preschools and public health authorities have a key role in connection with information and guidance of the parents. The information and guidance can be given in connection with conversations with the parents in preschool or health examinations. This will promote awareness of the importance of physical activity for health benefits, regardless of socioeconomic status, and prevent health inequalities.

However, as children are growing, they are exposed to various environments and are influenced by more than just their parents and closest family members. The choice of being in physical activity is therefore influenced by a person’s physical activity habits [32]. Children’s choice of activity might therefore become more apparent as parents gradually stop “babysitting” them, and they take more decisions on their own. This is supported in an exploratory, in-depth study conducted by Mulvihill, Rivers and Aggleton [34], where the majority of the children reported that more activities were carried out as a family unit at a young age, and that such activities naturally decreased as they got older. Therefore, it is of great importance that older children and adolescents get opportunities to be physically active in school after the natural urge to be active decreases. This can be done through planned and organized activities, and a physical environment that invites and stimulate the children and adolescents to be physically active. In this way, all children and adolescents will have the opportunity to develop a physically active lifestyle, regardless of their parents’ income and education level. In line with this, it is also important to create opportunities for physical activity in neighborhoods so there will be a low threshold, both practical and economical, for children and adolescents to be physically active.

However, neither the present study nor existing research on the relationship between parental socioeconomic status and preschool children’s physical activity, provides consistent findings. The contradiction between the present study’s results and results in other studies could be explained by several factors. It might be explained by the assumption that families with young children carry out activities as a family unit, as young children might have a greater need for “babysitting”. In contrast, a longitudinal study conducted by Anderssen et al. [61] indicate that levels of physical activity among young adults are not directly related to parental levels of physical activity, indicating that activities carried out as a family unit decreases as the child grows older.

After preschool during weekdays, there is only 3–4 h left of leisure time prior bedtime, which means that there is just a small amount of time for parents to be associated with the children’s physical activity in the midst of everyday activities. This could, however, have been different if parents spent more time with the children during weekdays. In addition, this is supported by the results in the present study, as it illustrates that the preschool children’s activity level at leisure differ between weekdays and weekends (Table 1), which is natural based on the time spent in leisure. Nevertheless, this could possibly be explained by the assumption that parents have more time and energy to engage in activities with their children on weekends. Furthermore, an interesting aspect of this, is the fact that leisure time during weekdays expands as children grow older, providing more time for being physically active.

Children’s and adolescents’ total amount of physical activity have been shown to decrease with age [15]. This corresponds to the indication illustrating that preschool children are initially physically active. It does also illustrate the assumption of children making gradually more of their own choices in terms of being physically active or not as they are growing older. However, children’s decisions might be profoundly associated with their parents. According to Dowda et al. [20], children’s participation in sports were related to family support, which in turn was significantly related to children’s MVPA. Research has also suggested that both preschool- and school-children’s participation in organized physical activity often are connected to their parents’ interest and support [62,63], indicating that there is a distinction between families who participate in sports and those who are not. From a health perspective, this underlines the importance of facilitating physical activity for all children in preschool, older children and adolescents in school to ensure that all children and adolescents meets the global recommendations for physical activity. Low-threshold activities in neighborhoods without requirements for equipment and costs should also be a priority for health authorities.

Nonetheless, it should be noted that the offer of organized sport might differ from cities to rural areas, as some areas are offered organized sport in preschool age, whereas other areas are not offered organized sport before school age. This might have had an impact on the present study, as the participants come from both cities and more rural areas.

The results in the present study, alongside with the non-consistent existing findings could also be explained by unreliable measurements. The field of research investigating the association between parents’ socioeconomic status and children’s physical activity level, independent of age, consist of various ways of measure both socioeconomic status and physical activity. Many studies involving children above preschool age, that report a positive association between socioeconomic status and children’s physical activity level, tend to use questionnaire as the only method to gather information on physical activity [23,35,36,37,38,39], which is considered as less reliable than objective measurements such as accelerometers.

Conflicting findings have also been found related to how socioeconomic status is measured, where the phenomena have been studied using only education [18,22,23] or income [24,25] as the socioeconomic status variable. Others have used both these variables [35,36,37,38] in order to explain parents’ socioeconomic status. However, it might be beneficial to look at the total household income instead of mothers and fathers’ income separately. This approach will allow more children to be included in the study, and the results will perhaps be more reliable in terms of the socioeconomic circumstances the child lives in. The total household income can be very unevenly distributed between parents, and although the income from one of the parents is relatively low, it is possible that the family socioeconomic status is high because the other parent has a high income.

## 5. Strengths and Limitations of the Study

This study has several strengths. Firstly, a major advantage includes that subjects’ physical activity were measured objectively using Actigraph GT1M accelerometers, which is validity- and reliability-tested for investigating the activity level and intensity of children between zero to five years old [46,47,48,49]. In addition, the specific type of accelerometer used in the present study corresponds well with energy expenditure in free-living activities [40]. Among other things, it filters out noises that are beyond human movement [13], such as vibration from transportation in motor vehicles [64]. Lastly, the present study does also include a more or less even gender distribution, which display the actual gender distribution in preschools. The sample included preschools independently of size and type of preschool, providing a representative sample.

In contrast, the present study does also possess several limitations. Among other things, the ability to generalize the findings is limited due to the uniqueness of the sample. To be included in the study the children had to be from two-parent families. This could be unfortunate, as some families only had one parent fill out the questionnaire and some children are raised without two parents. The fact that these children was excluded from the study, caused the response rate to be somehow low (43%), however, it is important to note that statistical analysis indicates that the drop out seems to be random.

Additionally, it is important to note some limitations with accelerometers. Perhaps the biggest disadvantage is that these instruments are known for their inability to capture PA during cycling when positioned at the hip [64]. This in unfortunate, as riding vehicles have been found to be an equipment that may lead to more minutes in MVPA for preschool children [65]. Furthermore, physical activities involving water were not included due the fact that the instruments were not water resistant. Based on this, results could be caused by a systematic bias in the physical activity measurements.

## 6. Conclusions

The results in the present study point to no significant relationship between the parental education and income, and their children’s physical activity level at leisure. Previous studies about parental socioeconomic status and preschool children’s physical activity level, have conflicting findings, while findings from studies among older children point to positive correlations. Findings in this study do not suggest a link between socioeconomic status and physical activity in this sample of children attending preschool full time in Norway. The emphasis on activity in the curriculum of kindergartens in Norway could be one explanation. A second possibility is that the variation in socioeconomic status was limited. Further research is needed to examine whether socioeconomic status is related to physical activity among older children in Norway or in young children who do not attend preschool full time or who live with a single parent.

## Figures and Tables

**Table 1:** Descriptive characteristics of children’s MVPA level at leisure.

	Boys (SD)	Girls (SD)	Total (SD)
Sample size (*n*) Mean age	125 4.3	119 4.2	244
Mean MVPA leisure time, weekdays	33.6 ± 12.6	30.8 ± 12.8	32.3 ± 12.8
Mean MVPA leisure time, weekend ^a^	69.4 ± 38.2	64.2 ± 33.4	73.5 ± 28.6

^a^ Two boys and four girls did not wear their accelerometer during the weekend.

**Table 2:** Descriptive characteristics of children’s MVPA level at leisure, according to the independent variables: mothers’ education level and annual income.

	Leisure Time Mean	Standard Derivation	*N* (*n* = 198)
Mothers’ education level Primary school	30.2	10.3	10
High school	34.2	13.6	68
College and/or university	30.0	11.4	120
Mothers’ income			
up to 500,000 NOK (50,000 Euros)	32.8	12.4	142
500,000–700,000 NOK (50,000–70,000 Euros)	28.1	10.9	38
>700,000 NOK (70,000 Euros)	28.4	14.0	12

**Table 3:** Descriptive characteristics of children’s MVPA level at leisure, according to the independent variables: fathers’ education level and annual income.

	Leisure Time Mean	Standard Deviation	N (*n* = 153)
Fathers’ education level Primary school	39.7	12.9	10
High school	32.7	12.1	76
College and/or university	31.5	13.6	67
Fathers’ income			
up to 500,000 NOK (50,000 Euros)	33.0	12.3	54
500,000–700,000 NOK (50,000–70,000 Euros)	33.3	14.2	57
>700,000 NOK (70,000 Euros)	30.7	12.5	40
